# Editorial: Emerging Chemical Risks for Human Health: Endocrine Disruption by Per- and Poly-Fluorinated Alkyl Substances (PFAS)

**DOI:** 10.3389/fendo.2021.813785

**Published:** 2022-01-12

**Authors:** Andrea Di Nisio, Maria-Jose Lopez-Espinosa, Carlo Foresta

**Affiliations:** ^1^Department of Medicine, Unit of Andrology and Reproduction Medicine, University of Padova, Padova, Italy; ^2^Epidemiology and Environmental Health Joint Research Unit, FISABIO–Universitat Jaume I–Universitat de València, Valencia, Spain; ^3^Spanish Consortium for Research on Epidemiology and Public Health (CIBERESP), Madrid, Spain; ^4^Faculty of Nursing and Chiropody, University of Valencia, Valencia, Spain

**Keywords:** endocrine disrupting chemicals, PFAS, endocrinology, translational medicine, reproduction, metabolism, neurodevelopment, puberty

Great attention has been paid in recent years to the harmful effects of various chemicals that interfere with hormonal function, collectively known as endocrine-disrupting chemicals (EDCs). Among EDCs, per- and poly-fluorinated alkyl substances (PFAS) are emerging chemicals raising health concerns worldwide. PFAS are a group of more than 4,700 man-made chemicals: perfluorooctanoic acid (PFOA) and perfluorooctane sulfonic acid (PFOS) represent the two most diffused and studied in this chemical class. PFAS are used in a wide variety of consumer products and industrial applications because of their unique chemical and physical properties, including oil and water repellence, temperature and chemical resistance, and surfactant properties. For these reasons, PFAS have been used in a wide variety of industrial and consumer products (e.g., firefighting foams, non-stick metal coatings, food packaging, cosmetics, textiles, photography, chrome plating, pesticides and pharmaceuticals). PFAS accumulate in humans, animals and the environment. This adds to the total burden of other chemicals to which people are exposed and increases the risk of health impacts. Of the relatively few well-studied PFAS, most are considered moderately to highly toxic, particularly for children’s development.

This Research Topic provides an overview of more recent clinical and basic insights about the possible impact of PFAS on human health and expresses the opinions of experts from different areas of medicine and biology who have expanded the field with their recent discoveries. The Research Topic contains 4 contributions, including two original research articles and two reviews.

Incident diabetes and increased fasting glucose have been variably described as clinical outcomes associated with PFAS exposure, largely depending on the molecule type, level of exposure, age, underlying comorbidities, developmental period of exposure and geographic area. However, diabetes-related cardiovascular mortality has been recently claimed as a major complication associated with environmental exposure to PFAS. The review by Meneguzzi et al. analyzed the epidemiological and experimental studies exploring the relationship between exposure to PFAS and thromboembolic cardiovascular disease. The authors also present the recent experimental evidence indicating how new-generation PFAS could represent a possible risk factor for thromboembolic events. This review offers mechanistic support to the hypothesis of the implication of platelet-centered mechanisms in the increase of cardiovascular events observed in populations chronically exposed to PFAS.

Major liver toxicity of PFAS has been also reported: results from preclinical research clarified possible mechanisms by which PFOA can interfere with the regulation of hepatic glucose metabolism (De Toni et al.). Using of HepG2 cells, an *in vitro* model human hepatocyte, the authors evaluated glycogen synthesis, glucose uptake and Glut-4 glucose transporter translocation upon PFOA, and insulin exposure. Through the use of an *in vitro* approach, in this study the authors provided evidence that the acute exposure of human hepatocytes to PFOA was associated with the impairment of insulin receptor (InsR) signaling, resulting in reduced glucose uptake and reduced glycogen synthesis. From a mechanistic point of view, this effect likely relies on the interference of PFOA with the InsR at membrane level, resulting in early uncoupling between InsR activation/autophosphorylation and downstream signal transduction. The conclusion of this paper underlined the importance of pre-clinical data providing more direct inferences to changes in biological activity, allowing the screening of the direct effects of chemicals on a specific tissue or cell.

Early PFAS exposure might also affect growth, adiposity and pubertal development during the fetal stage and the first years of life, being these outcomes the aim of the review published by Lee et al. The published literature up to date led to the judgment by the authors that there was some consistency of a possible impairment of fetal growth and child adiposity due to prenatal PFAS exposure. However, the existent literature on pubertal development and child growth was considered by authors somewhat limited and inconclusive to draw a conclusion from, with further studies warranted. Although the possible biological mechanisms underlying the potential effects of PFAS on the above-mentioned outcomes are still not well understood, Lee et al. suggested several possible modes of action (specifically, PFAS-induced PPAR activation, alteration of sex and thyroid hormone biosynthesis and metabolism due to exposure to these contaminants, and the PFAS ability of acting as estrogenic or anti-androgenic substances).

PFAS have also been related to neurodevelopmental toxicity in animal studies, but results from human research remain inconclusive. These inconsistencies might be explained to some extent by the different methodological approaches used in the articles as well as due to the complexity of the functioning of the brain. In this sense, the development of the central nervous system is an extremely complex process and toxic exposure might disrupt multiple biological pathways affecting the entire neurodevelopmental spectrum with long-lasting consequences in academic and career success, mental well-being, and quality of life. Yu et al. tried in their article to shed light upon the possible association between PFAS and neurodevelopment by using a biomarker of brain development and functioning: the brain-derived neurotrophic factor (BDNF). They found a positive association between early pregnancy exposure to perfluorohexanesulfonic acid (PFHxS) and cord BDNF levels, being stronger for boys. The authors concluded that there is need for further studies to clarify how PFAS might target brain development as well as the possible sex-specific mechanisms underlay the increased vulnerability to PFAS exposure of one sex or the other.

To date, any conclusion about cause–effect relationships is hindered by the cross-sectional design of most of the published studies and the large variability of different PFAS chemicals considered and respective exposure levels. Furthermore, despite the adjustment for possible confounding factors in different studies, other unmeasured confounders could have influenced the associations under investigation. Obviously, the best evidence of an adverse effect of PFAS on human health would be provided by longitudinal analyses, assessing clinically relevant endpoints.

While the latter represents a real challenge for future research, we would like to express our sincere gratitude to all authors and referees for their contribution to this Research Topic summarizing the multidisciplinary and collaborative efforts which in recent years have helped shed some light on a topic yet to be largely investigated.

## Author Contributions

AN, M-JL-E, and CF conceived, wrote, and approved the final version of the manuscript.

## Conflict of Interest

The authors declare that the research was conducted in the absence of any commercial or financial relationships that could be construed as a potential conflict of interest.

## Publisher’s Note

All claims expressed in this article are solely those of the authors and do not necessarily represent those of their affiliated organizations, or those of the publisher, the editors and the reviewers. Any product that may be evaluated in this article, or claim that may be made by its manufacturer, is not guaranteed or endorsed by the publisher.

